# The Role of Natural Products on Diabetes Mellitus Treatment: A Systematic Review of Randomized Controlled Trials

**DOI:** 10.3390/pharmaceutics14010101

**Published:** 2022-01-02

**Authors:** Lucía Vivó-Barrachina, María José Rojas-Chacón, Rocío Navarro-Salazar, Victoria Belda-Sanchis, Javier Pérez-Murillo, Alicia Peiró-Puig, Mariana Herran-González, Marcelino Pérez-Bermejo

**Affiliations:** 1 School of Medicine and Health Sciences, Department of Nutrition, Catholic University of Valencia San Vicente Mártir, C/Quevedo nº 2, 46001 Valencia, Spain; luviba@mail.ucv.es (L.V.-B.); mariarojaas96@mail.ucv.es (M.J.R.-C.); rocioinmaculada.navarro@mail.ucv.es (R.N.-S.); victoria.belda@mail.ucv.es (V.B.-S.); javierperezmurillo@mail.ucv.es (J.P.-M.); alicia.peiro@mail.ucv.es (A.P.-P.); mariana.herran@mail.ucv.es (M.H.-G.); 2 SONEV Research Group, School of Medicine and Health Sciences, Catholic University of Valencia San Vicente Mártir, C/Quevedo nº 2, 46001 Valencia, Spain

**Keywords:** diabetes mellitus, type 2, biological products, plants, antioxidants, therapeutics, natural products, phenols

## Abstract

The present study was carried out to relate the role of natural products in the metabolism of an increasingly prevalent disease, type 2 diabetes mellitus. At present, in addition to the pharmacological resources, an attempt is being made to treat diabetes mellitus with natural products. We carried out a systematic review of studies focusing on the role of natural products on diabetes mellitus treatment. The bibliographic search was done through Medline (Pubmed) and Web of Science. From 193 records, the title and summary of each were examined according to the criteria and whether they met the selection criteria. A total of 15 articles were included; after reviewing the literature, it is apparent that the concept of natural products is ambiguous as no clear boundary has been established between what is natural and what is synthetic, therefore we feel that a more explicit definition of the concept of “natural product” is needed. Gut microbiota is a promising therapeutic target in the treatment of diabetes. Therefore, it would be necessary to work on the relationship between the microbiome and the benefits in the treatment of diabetes mellitus. Treatment based solely on these natural products is not currently recommended as more studies are needed.

## 1. Introduction

The rapid development of society over the 21st century has brought about a complete change in lifestyle of the population in both a positive and negative way; new risk factors have emerged that have conditioned an increase in the prevalence of chronic diseases worldwide, such as type 2 diabetes mellitus (T2D), which in turn increases the morbidity and mortality of the world population [1].

Type 2 diabetes is a multifactorial metabolic pathology. The WHO puts its prevalence at over 422 million subjects, and a total of 1.6 million die per year. It is estimated that eight out of every 1000 inhabitants suffer from it and its prevalence increases in the elderly; however, it is becoming increasingly common in children and adolescents [2]. The constant increase in the population diagnosed with T2D, together with its associated complications, makes it a first-order healthcare and economic problem whose impact is reflected in its treatment and complications that cause poor quality of life. In fact, annual healthcare costs are calculated to be between 121.97–141.6 million euros, which is why it is estimated that people with T2D generate twice the healthcare costs of persons who do not suffer from this pathology. Type 2 diabetes is currently deemed one of the pandemics of the 21st century [1,2].

The development of T2D is frequently associated with a combination of a failure in the functioning of the β cells of the pancreas and insulin resistance in various target tissues, such as liver, muscle and adipocytes [3]. Healthy β cells compensate for insulin resistance with an increase in insulin secretion, but a failure in this compensatory mechanism leads to glucose intolerance. Once hyperglycemia occurs, β-cell function deteriorates and insulin resistance worsens, a process known as glucose toxicity [4]. Prior to the onset of diabetes, there is a stage known as pre-diabetes; as it progresses, alterations occur in the cells of the pancreas that make up the islets of Langerhans [1,2].

A sedentary lifestyle, being overweight and malnutrition generate an increase in the production of different reactive oxygen species which produce a chronic state of oxidative stress. This alters the secretion of insulin by the pancreas and the action of hormones in target cells, generating a greater risk of macro- and microvascular complications [5]. There is scientific evidence showing that β cells have very low levels of antioxidant enzymes compared to other tissues and there lies their high vulnerability to oxidative stress. As mentioned, T2D is a chronic disease that combines different metabolic disorders that coexist in a positive feedback loop guided by inflammation. Therefore, reducing and fighting inflammation, as well as oxidative stress, is one of the main therapeutic objectives [6].

There are numerous chemical compounds included in drugs that help control blood glucose levels, such as oral antidiabetics and preloaded insulins. At present, in addition to these pharmacological resources, an attempt is being made to treat this pathology with natural products [7]. Natural products started being used to control blood glucose levels ever since they took center stage in experimental investigations; some of these plants are *Bauhinia forficata*, *Cecropia obtusifolia* (Bertol), *Equisetum myriochaetum* and *Cucurbita ficifolia* bouche, among others [6,7,8]. Treatment using them has a notable local connotation, since natural products generally vary depending on the country and its culture. An example of this can be seen in Latin America, where *Bauhinia forficata* is more often used, while in Sri Lanka *Senna auriculata* (L.) is used more routinely [9]. Other products, such as alfalfa, *Ginkgo biloba*, ginseng and turmeric are found more frequently. Familiarity with the properties of plants and scientific evidence are the starting point for using this type of product in the treatment of chronic diseases such as diabetes [10].

Current drugs for treatment of diabetes employ antidiabetic mechanisms of action that include the inhibition of alpha-glucosidase and alpha-amylase in the digestive tract, decreasing the uptake of glucose through its transporters by stimulating the release of insulin [11]. However, there are also many others, especially the most recent ones, which try to imitate the mechanism of action of natural products by incorporating several of their active principles. At present, the most common drug on the market for glucose control is metformin, which comes from *Galega officinalis*, a guanidine-rich plant used in European folk medicine [12,13]. In an attempt to find a similar drug, the study of *Ginkgo biloba*, ginseng and turmeric, among many others, has been implemented. This shows the true importance of the investigation of plants that can be sources of new compounds with clinical activities for the treatment of chronic diseases, such as diabetes [10,13].

Although reviews on the use of natural products in T2D have previously been published [14,15,16], these are works that analyze in depth the therapeutic effects and biochemical mechanisms of different natural products against T2D, reporting that most analyzed studies provide preliminary or inadequately documented results and that is necessary to continue working on their research and development. This review, focused exclusively on randomized clinical trials, tries to delve deeper into the work that has been carried out in recent years on the application of natural products to treat the disease.

## 2. Search Methodology

**Figure 1:**
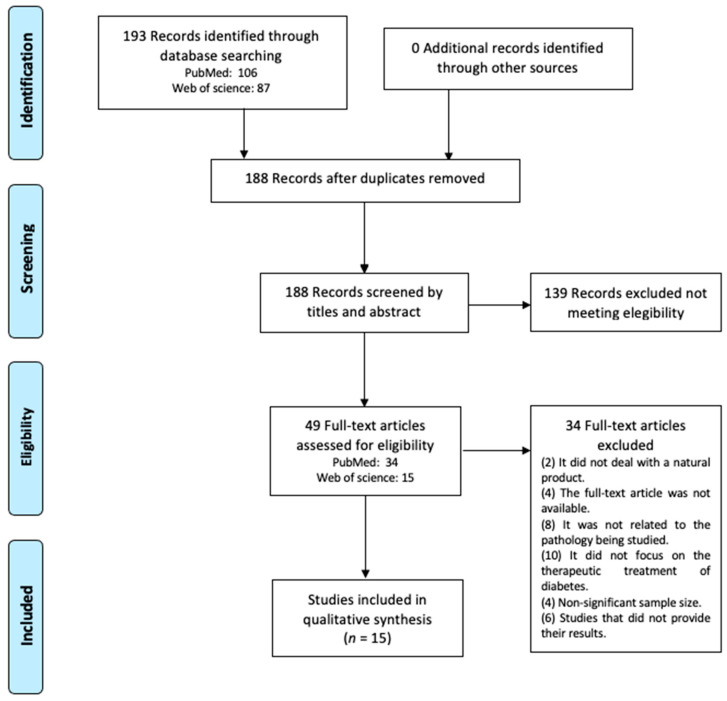
PRISMA flowchart of study selection process.

This systematic review was conducted in accordance with the criteria set out in the Preferred Reporting Items for Systematic Reviews and Meta-Analysis (PRISMA) [17]. The literature search was carried out in PubMed and Web of Science. The search strategy was carried out by combining the terms “Diabetes Mellitus”, “Type 2”, “Biological Products”, “Antioxidants”, “Plants”, “Therapeutics” and “Phenols”, combined with each other using Boolean operators. Randomized controlled trials from within the last five years were selected, giving a total of 15 to review. The flowchart in Figure 1 details the search and selection process. 

The Joanna Briggs Institute (JBI) [18] checklist for randomized controlled trials was used to assess study design and quality. One point was ascribed to each criterion achieved on the checklist. The quality of the studies was rated as a percentage of the total available points on the checklist.

## 3. Results

As shown in Figure 1, the literature search identified 193 records. After removing duplicates and screening titles and abstracts, 49 articles were selected for full-text review, of which 15 studies met the inclusion criteria [19,20,21,22,23,24,25,26,27,28,29,30,31,32,33]. Table 1 shows the main characteristics of each study. Table 2 shows the quality assessment of the studies.

## 4. Discussion

The present study was carried out to relate the role of natural products in the metabolism of an increasingly prevalent disease, type 2 diabetes mellitus (T2D) [1,2]. After reviewing the literature, it is apparent that the concept of natural products is ambiguous as no clear boundary has been established between what is natural and what is synthetic, since a product can be synthesized from a natural extract. Metformin, for example, is obtained from a derivative of the guanidines of the French lilac, *Galega officinalis* [34]. Insulin can also be synthesized from bacterium *E. coli*, building or transforming an analogue of human insulin [35]. This has made it difficult to search bibliographies as it has not been possible to delimit some keywords to make an exact search.

The studies analyzed do not show enough scientific evidence to use the methods investigated in a population. More research is required to be able to observe possible side effects caused, in the short and long term, by the use of these treatments with natural products at the individual and collective level [21,23,26,27,28,32,33]. Some studies [32,33,36,37] indicate that more research is required to determine evidence of beneficial effects through the use of natural products.

Studies show that natural products have more than one beneficial effect in addition to being insulin-sensitizing or hypoglycemic as they can be anti-inflammatory, antioxidant and cholesterol-lowering [22,23,24,25,27,32]. Zakerkish et al. [27] used Iranian propolis collected from beehives and its use was found to provide various beneficial effects in patients with T2D. These results indicate that a promising treatment can be achieved with long-term studies since the use of natural products has good potential.

Insulin (SAR-Asp) has been shown to have an effective glycemic control effect very similar to the different insulins found on the market [19]. It is important to evaluate the significant effect of this insulin to implement a longer study time. In another study, the insulin-sensitizing effect of *Scutellaria baicalensis* (SB) was proposed as an adjunct to metformin in the treatment of type 2 diabetic patients and also indicates that SB improves metabolism and the number of microbial taxa, which suggests that this treatment can improve glucose metabolism through modulation of the gut microbiota in patients with T2D [20].

The studies analyzed in this work presented some difficulties when deciding whether or not they should be part of the bibliographic review. As mentioned above, this was due to the indistinct boundary between the concepts of natural and synthetic products as well as the border between treatment and patient improvement. Another limitation is that the analysis of the action of natural products is restricted to an adjuvant action together with drugs in current use [20,23], which makes it difficult to define the benefits of natural compounds. However, it can be stated that as an adjunct to metformin, Gingko biloba extract [23] as well as treatment with *Scutellaria baicalensis* extract [20] present benefits compared to metformin therapy alone [20,23]. Among the outstanding natural products in the treatment for T2D, we can highlight *Ginkgo biloba* extract as an adjuvant to Metformin [23], pinitol [24], propolis [27], live probiotic L. Reuteri ADR-1 and heat-killed probiotic L. reuteri ADR-3 [29], mixed berries [30], a Chinese plant extract [22] and resveratrol [32].

A limitation of this review is that the results of the studies were obtained over a short period of time, from three to six months, so these results may be affected if an increase in the temporality of the treatments is protocolized [23,24,25,26,27,28,34]. Within the application of natural products as a treatment for patients with T2D, there are single products that can have effects at different levels, that is, a natural product can generate a modulating effect on glucose and lipid metabolism hypoglycemia, hypolipidemia, modulation of the microbiota and anti-inflammatory and antioxidant agents [22,23,24,27,29,30,32]. Their multiple applicability and their effect in the short and long term must be evaluated and taken into account; consequently, studies are necessary in which the greatest number of these aspects are measured so that more significant results can be obtained. 

The studies analyzed show the close relationship between the diversity of microorganisms in the intestinal microbiota and the functioning and metabolism of the host, as well as linking certain microorganisms with specific effects [36,38,39]. The studies by Tong et al. [22], Na Rae Shin et al. [20] and Ming-Chia et al. [29] show promising benefits for the treatment of T2D by increasing diversity and certain species in the intestinal microbiota, making it a promising therapeutic target for the disease in question. Specifically, it would be interesting to study in depth the benefits of the increase in microbiota of butyrate-producing species as well as *Blautia* spp. and *Faecalibacterium* spp. since they show an improvement in carbohydrate and lipid homeostasis [22]. By contrast, other studies reported no benefit for the treatment of T2D [21,26,28,32].

In general, it can be concluded that the most notable benefits of treatments and protocols using natural products in our review studies are the improvement in insulin resistance of the subjects [19,22,27,29,31], improvement in biochemical parameters of glycosylated hemoglobin [23,25,27,29], improvement in the lipid profile in general [22,23,27,29,30] and a reduction in glucose levels in pre-prandial blood [20,23].

In this context, it is also a source of discussion that the presence of environmental factors and factors specific to individuals can interfere with the results obtained, since, for example, when carrying out a therapy with natural products accompanied by adjuvant drugs, it is difficult to determine if the effect obtained is due to the use of the natural treatment or the drug. In addition, the presence of toxic habits such as an unbalanced diet, among other aspects, can be the cause of the possible negative effects that are associated with a natural product. The individuality of the patient makes the response to the treatments totally different, since individual factors, such as lifestyle, eating habits, genetic factors, underlying pathologies and treatments, can lead to totally different results. That is why when considering this type of study, it is necessary to evaluate the personal factor that characterizes each individual. This fact, together with the small sample size, can interfere with how results can be extrapolated to a larger population.

## 5. Conclusions

The purpose of this study was to analyze the effect of natural products as a therapy in T2D. After conducting this systematic review, we feel that a more explicit definition of both the concept of “natural product” and that of “treatment” is needed. This would make it easier to select and filter different studies based on whether they focus on therapeutic treatment, nutritional treatment or quality of life improvement.

Gut microbiota is a promising therapeutic target in the treatment of diabetes. We also observe that there is a correlation between the presence of specific species and therapeutic properties. Therefore, it would be necessary to work on the relationship between the microbiome and the benefits in the treatment of T2D.

Natural products that improve comorbidities related to metabolic syndrome and T2D, such as oxidative stress and chronic systemic inflammation, have beneficial effects on patients. However, treatment based solely on these natural products is not currently recommended as more studies are needed. Nonetheless, natural products favoring T2D treatment may be a promising adjunct to current therapies. However, the present review shows some limitations in the protocols of the different studies that make drawing conclusions difficult, which is why studies with more specific protocols are needed, such as long-term vision and larger samples to confirm the benefits and to standardize the results obtained.

## Figures and Tables

**Table 1:** Main characteristics of each study analyzed.

Ref.	Year	Type of Study	Sample	Results	Conclusions
[19]	2020	Randomized controlled trial	580	HbA1c improved similarly in both treatment groups. Changes in fasting plasma glucose and plasma glucose profile and insulin doses were similar in both groups.	This study concludes that SAR-Asp was well tolerated and demonstrated effective glycemic control with a safety and immunogenicity profile similar to that of commercially available insulin aspart formulations in people with diabetes treated for 26 weeks.
[20]	2020	Randomized controlled trial	17	BS with metformin treatment improved glucose tolerance and the expression of inflammatory markers in patients with T2D. Furthermore, a considerable number of bacterial taxa were correlated with clinical markers, especially with blood glucose during OGTT. Furthermore, metagenomic data indicated that the metabolism of seleno compounds increased after treatment with BS.	The results obtained suggest that treatment with BS and metformin can improve glucose metabolism through modulation of the intestinal microbiota in patients with T2D.
[21]	2018	Randomized controlled trial	39	Blood pressure decreased in patients treated with extracts of J. regia. It also showed a significant decrease in body mass. Thus, hydroalcoholic extracts of J. regia do not produce beneficial effects in patients with T2D.	We cannot conclude that the extracts obtained from walnut leaves may be a future treatment for T2D.
[22]	2018	Randomized controlled trial	450	Only AMC improved insulin resistance and triglyceride levels. Both metformin and AMC treatments improved blood glucose, glycosylated hemoglobin, cellular function of beta cells; they both increased gut microbiota diversity, thereby decreasing the number of subjects in the groups related to the pathogenesis of the disease. AMC showed a greater modulating effect on the microbiota as well as an increase in the concentration of beneficial microorganisms for the treatment of T2D.	This study suggests that the beneficial effects of metformin and AMC are due to the modification in the intestinal microbiota. It was possible to correlate the increase in *Blautia spp.* and *Faecalibacterium spp.* with the improvement in both lipid and glucose homeostasis. The results show that the gut microbiota is a promising therapeutic target for diabetes. It will be interesting for future studies on the diabetes treatment capacity of mangiferin and berberine.
[23]	2018	Randomized controlled trial	47	In the placebo group, no significant decrease in the concentration of glycated hemoglobin was observed. On the other hand, it was in the group treated with GKB and the same for fasting blood glucose. Regarding the decrease in insulin levels in the treatment group, no significant differences were observed. Body mass index, visceral adiposity and hip circumference decreased in GKB patients in contrast to the group treated only with metformin or placebo.	The results therefore show that *Ginkgo biloba* extract is a promising adjuvant to metformin. However, more studies are needed to analyze its long-term effects as well as a study with a larger number of participants.
[24]	2018	Randomized controlled trial	118	Six weeks of intake of pinitol-enriched beverages resulted in a significant increase in two proteins involved in the insulin secretion pathway, the acid labile subunit of insulin-like growth factor and complement C4A in glucose intolerant subjects, but not in healthy volunteers.	This study concluded that substituting a common sugar source (such as sucrose) for a natural drink enriched with pinitol in subjects with glucose intolerance could benefit glucose metabolism by reducing and stimulating insulin secretion. This mechanism may play an important role in the prevention of insulin resistance and the progression of diabetes.
[25]	2017	Randomized controlled trial	60	The mean values of fasting blood glucose, glycated hemoglobin and triglycerides in the group of herbal medicines, were significantly lower than the values of the placebo group.	The study shows a potential antihyperglycemic and triglyceride-reducing effect through the use of a mix of silymarin, nettle and olibanum extracts. It caused a significant reduction in glycated hemoglobin, triglycerides, and fasting blood glucose. It did not reduce cholesterol or blood pressure. It stated that other hypoglycemic agents should be used to confirm these effects.
[26]	2019	Randomized controlled trial	81	The HbA1C and the fasting blood glucose remained the same. Insulin sensitivity was significantly improved in the group with the curcumin supplement compared to the double placebo group. The triglyceride level increased in the double placebo group and the group with the curcumin and LCn-3 PUFA supplementation decreased triglyceride levels.	This study does not provide evidence of a curcumin and LCn-3 PUFA-based supplementation. This can be due to various factors such as the study population, balance in the groups or interactions between bioactives. A curcumin-based supplementation does have a positive effect on improving insulin sensitivity. It is believed that these results may indicate that a better strategy can be carried out to reduce risk factors in the progression of T2D.
[27]	2019	Randomized controlled trial	94	The effect of propolis on glucose metabolism shows that after the intervention, the mean HbA1C, 2hpp FBS and insulin decreased significantly compared to the placebo group. It also significantly decreases HOMA-IR and HOMA-β. It is suggested that glycemic control is related to the intestinal reduction of carbohydrates and increases the level of glycolysis and the use of glucose in the liver through increasing the absorption of glucose by peripheral tissue by activating the glucose-sensitive transporter.	Iranian propolis has effects on post-pandial blood glucose, serum insulin, and insulin resistance. The study also mentions the decrease in inflammatory cytokines, associated with oxidative stress and chronic inflammation. This pathogenesis is related to T2D.
[28]	2017	Randomized controlled trial	39	After the 8-week intervention supplemented with the three grams of cinnamon, there were no changes or findings on the effects that cinnamon may have.	This study was designed to evaluate whether cinnamon intake influenced glycemic markers, glycation end products, and inflammatory indicators in patients with T2D.
[29]	2018	Randomized controlled trial	74	The study analyzed the cytokines, glutathione, peroxidase, HbA1c and the fecal microbiome composition. Probiotics being killed by heat have shown to positively affect immunodeficient patients. HbA1c levels were significantly reduced in patients taking L. reuteri ADR-1in the tests taken at the visits 2, 3 and 4. In this sense, the patients were able to maintain a stable HbA1c level for 3 months after the intervention. A decrease in blood lipids was observed: cholesterol, free fatty acids and LDL in groups with ADR-1, although only cholesterol was significant. In the ADR-3 group, a significant reduction in arterial pressure and loss of body weight was observed. The inflammatory cytokines IL-1Beta showed a significant reduction in the group with ADR-3. Regarding the microbiome, L. reuteri increased significantly in the group with ADR-1. A significant increase in bifidobacterium was also observed in the ADR-1 group.	The conclusion of the results suggest that the reduced levels of HbA1c are positively affected by L. reuteri after the regulated consumption of ADR-1 and ADR-3 and that changes in the microbiome are the result of its ingestion. These changes should have a study conducted examining regulating blood sugar levels and further TD2 complications.
[30]	2019	Randomized controlled trial	52	The study showed that HbA1c levels did not vary significantly between the two groups: patients taking the supplement decreased significantly in terms of TG compared to the placebo group after 3–6 months of taking the supplement. In the group taking the supplement, BMI increased and adiponectin decreased, probably since curcumin tends to increase appetite, although the mechanism is not clear. Leptin also decreased, probably due to the improvement in the leptin resistance.	The study had a limitation which was the small sample size and the short duration of the intervention. We would have to make a further study to reach a conclusion regarding the beneficial effects of curcumin on adiponectin and antioxidative LDL in patients with T2D. However, the study showed that curcumin inhibits the increase in oxidative LDL in patients with T2D, so it can be used to prevent cardiovascular diseases and common diabetes complications.
[31]	2019	Randomized controlled trial	36	The results of the study show that when in treatment, patients were asked to eat a high glucose meal in which blood samples were taken. Insulin levels were high and serum unesterified fatty acids were low. The berries showed an improvement in serum and insulin value close to the statistical significance. There was a significant reduction in the insulin response of the patients who consumed berries compared to those consuming gelatin.	The effect of each kind of berry in terms of glucoregulatory powers cannot be determined possibly due to the variability between their inclusion in the samples given to the patients during the study. Some berries have a more potent effect and this effect was diluted because of their mixture. Evidence has shown that berries have both short- and long-term effects, but further investigation might be needed to conclude these results.
[32]	2016	Randomized controlled trial	14	Some previous studies have shown an increase of the release of GLP-1 in diabetic mice. However, in this study, a dose of 500 mg of resveratrol administered twice a day for 5 weeks showed no effect on GLP-1 secretion, glycemic control, gastric emptying and body weight and did not suppress energy intake on type 2 diabetic humans	In total, 14 subjects (10 men and 4 women) with type 2 diabetes, managed only by diet, showed no effects after 5 weeks of resveratrol administration compared to the placebo group. It is worth bearing in mind the short duration of the study, the lack of obese subjects and the small sample size.
[33]	2017	Randomized controlled trial	86	An herbal combination capsule of 600 mg was composed of Terminalia chebula fruit extract (200 mg), Commiphora mukul (200 mg) and Commiphora myrrha oleo-gum-resin (200 mg). The participants were randomly assigned to either herbal combination or placebo group. The capsule was taken 3 times a day and showed an improvement of glycemic control, total cholesterol and low-density lipoprotein cholesterol. Moreover, it increased high-density lipoprotein cholesterol levels.	Eighty-six hyperlipidemic type 2 diabetic women between 40 and 60 years, fasting serum glucose levels between 150 and 180 mg/dL; blood glycosylated hemoglobin levels between 7.5% and 8.5%; low-density lipoprotein cholesterol > 100 mg/dL; and daily oral intake of not more than 10 mg glyburide and 1000 mg metformin at maximum were included in the study.The herbal combination was well tolerated and did not cause any hepatic, renal or other adverse effects. However, it is worth bearing in mind the short duration of the study and the different durations of the study between participants. The actual mechanism of drug action remains unknown at the moment. More studies are necessary to ensure the safety and effectiveness of the compounds.

Abbreviations: HbA1c: Glycated Hemoglobin. SAR-Asp: Insulin Aspart Biosimilar/follow-on biologic product SAR341402; BS: Scutellaria Baicalensis. T2D: Type 2 Diabetes. OGTT: Oral Glucose Tolerance Test. AMC: Traditional Chinese Herbal Formula (Extract of the dry corolla of A. manihot). GKB: *Ginkgo biloba* Extract. C4A: Complement C4A. LCn-3 PUFA: Long-Chain n-3 Polyunsaturated Fatty Acid. 2hpp: 2-h post prandia. FBS: Fasting Blood Sugar. HOMA-IR: Homeostasis Model Assessment-Insulin Resistance. HOMA-β: Homeostasis Model Assessment of β-cell function. LDL: Low-Density Lipoprotein. TG: Triglycerides. BMI: Body Mass Index. GLP-1: Glucagon-Like Peptide 1.

**Table 2:** Studies appraised using the Joanna Briggs Institute critical appraisal checklist for randomized controlled trials.

Study	Was True Randomization Used for Assignment of Participants to Treatment Groups?	Was Allocation to Treatment Groups Concealed?	Were Treatment Groups Similar at the Baseline?	Were Participants Blind to Treatment Assignment?	Were Those Delivering Treatment Blind to Treatment Assignment?	Were Outcomes Assessors Blind to Treatment Assignment?	Were Treatment Groups Treated Identically Other Than the Intervention of Interest?	Was Follow up Complete and If Not, Were Differences between Groups in Terms of Their Follow up Adequately Described and Analyzed?	Were Participants Analyzed in the Groups to Which They Were Randomized?	Were Outcomes Measured in the Same Way for Treatment Groups?	Were Outcomes Measured in a Reliable Way?	Was Appropriate Statistical Analysis Used?	Was the Trial Design Appropriate, and Any Deviations from the Standard RCT Design (Individual Randomization, Parallel Groups) Accounted for in the Conduct and Analysis of the Trial?	Score out of 13 (100%)
Garg et al., 2020 [19]	Y	Y	Y	N	U	Y	Y	U	Y	Y	Y	Y	Y	76.90%
Shin et al., 2020 [20]	Y	Y	Y	Y	Y	Y	Y	Y	Y	Y	Y	Y	Y	100%
Rabiie et al., 2018 [21]	Y	Y	Y	Y	U	Y	Y	Y	Y	Y	Y	Y	Y	92.30%
Tong et al., 2018 [22]	Y	Y	Y	U	U	U	Y	Y	N	Y	Y	Y	Y	69.20%
Aziz et al., 2018 [23]	Y	Y	Y	Y	Y	U	Y	Y	Y	Y	Y	Y	Y	92.30%
Lambert et al., 2018 [24]	Y	Y	Y	Y	Y	U	Y	Y	Y	Y	Y	Y	Y	92.30%
Khalili et al., 2017 [25]	Y	Y	Y	Y	Y	U	Y	Y	Y	Y	Y	Y	Y	92.30%
Thota RN et al., 2019 [26]	Y	Y	Y	Y	Y	U	Y	Y	Y	Y	Y	Y	Y	92.30%
Zakerkihs et al., 2019 [27]	Y	Y	Y	Y	Y	U	Y	Y	Y	Y	Y	Y	Y	92.30%
Talaei et al., 2017 [28]	Y	Y	Y	Y	Y	U	Y	Y	Y	Y	Y	Y	Y	92.30%
Hsieh et al., 2018 [29]	Y	Y	Y	Y	Y	Y	Y	Y	Y	Y	Y	Y	Y	100%
Funamoto et al., 2019 [30]	Y	Y	Y	Y	Y	Y	Y	Y	Y	Y	Y	Y	Y	100%
Solverson et al., 2019 [31]	Y	Y	Y	Y	Y	Y	Y	Y	Y	Y	Y	Y	Y	100%
Thazhath et al., 2016 [32]	Y	Y	Y	Y	Y	U	Y	Y	U	Y	Y	Y	Y	84.60%
Shokoohi et al., 2017 [33]	Y	Y	Y	Y	Y	U	Y	Y	Y	Y	Y	Y	Y	92.30%

Y = Yes. N = No. U = Unclear.
